# Raman Mapping Analysis of Graphene-Integrated Silicon Micro-Ring Resonators

**DOI:** 10.1186/s11671-017-2374-4

**Published:** 2017-11-22

**Authors:** Siham M. Hussein, Iain F. Crowe, Nick Clark, Milan Milosevic, Aravind Vijayaraghavan, Frederic Y. Gardes, Goran Z. Mashanovich, Matthew P. Halsall

**Affiliations:** 1 Photon Science Institute and School of Electrical and Electronic Engineering, University of Manchester, Manchester, M13 9PL UK; 2 School of Materials and the National Graphene Institute, University of Manchester, Manchester, M13 9PL UK; 3 Optoelectronics Research Centre, University of Southampton, Southampon, SO17 1BJ UK

**Keywords:** Graphene, Silicon photonics, Raman

## Abstract

We present a Raman mapping study of monolayer graphene G and 2D bands, after integration on silicon strip-waveguide-based micro-ring resonators (MRRs) to characterize the effects of the graphene transfer processes on its structural and optoelectronic properties. Analysis of the Raman G and 2D peak positions and relative intensities reveal that the graphene is electrically intrinsic where it is suspended over the MRR but is moderately hole-doped where it sits on top of the waveguide structure. This is suggestive of Fermi level ‘pinning’ at the graphene-silicon heterogeneous interface, and we estimate that the Fermi level shifts *down* by approximately 0.2 eV from its intrinsic value, with a corresponding peak hole concentration of ~ 3 × 10^12^ cm^−2^. We attribute variations in observed G peak asymmetry to a combination of a ‘stiffening’ of the *E*_2g_ optical phonon where the graphene is supported by the underlying MRR waveguide structure, as a result of this increased hole concentration, and a lowering of the degeneracy of the same mode as a result of localized out-of-plane ‘wrinkling’ (curvature effect), where the graphene is suspended. Examination of graphene integrated with two different MRR devices, one with radii of curvature *r* = 10 μm and the other with *r* = 20 μm, indicates that the device geometry has no measureable effect on the level of doping.

## Background

Integration with the silicon photonics platform is where graphene could have the largest impact, in applications such as photo-detection, optical modulation and biochemical sensing, thanks to the potential for CMOS back-end-of-line mass fabrication at relatively low cost [1]. In fact, research in this area is already now becoming established [2, 3], but in order to realize high performance devices, the graphene transfer process should be optimized and any processing-related modifications to graphene’s mechanical and electronic properties need to be properly characterized and understood. For instance, it is widely known that graphene-integrated silicon (and other) substrates tend to yield a significant amount of process contaminants and defects related to the heterogeneous material bonding, which can affect device quality at the junction between the two materials. Changes in the graphene band structure as a result of strain and unintentional doping at these interfaces can show up in the Raman scattering signatures, through changes in peak position, width, asymmetry and relative peak intensities. Raman spectroscopy has been used as a sensitive tool to evaluate graphene’s electronic and vibrational properties [4] including strain [5], doping level [6], defect density [7] and edge structure [8], although the effects of these can be difficult to separate from those influenced by the substrate. The intensity, width, shift rate and splitting of the graphene Raman peaks with strain and *p*- and *n*-type doping have already been reported [5, 9–11].

Graphene exhibits three principal Raman scattering peaks, each with a distinct physical origin: the doubly resonant (DR) D peak appears around 1350 cm^−1^ [12] and is related to disorder, generally, meaning that its appearance and relative intensity are often used as a measure of transferred material quality (i.e. it is weak or absent in high quality, pristine material). The other two main peaks are the G peak, which is derived from graphitic in-plane scattering of zone centre phonons and is located around 1580 cm^−1^ [8, 12], and the 2D peak (second order of the D peak), which appears around 2700 cm^−1^ [13]. Despite its relationship with the D peak, the 2D peak is strong in high quality, pristine graphene (i.e. when the D peak is absent) due to the fact that it satisfies the fundamental selection rule (*q* = 0) specifically by an electron-phonon DR scattering process, whereas the D peak requires highly localized electron-defect scattering to conserve momentum [12, 14–16]. The shape, intensity and positions of the G and 2D peaks allow determination of the number of graphene layers as well as any inherent strain and the presence of excess carriers in the material to be discerned [8, 13].

Graphene integration with the silicon photonics platform is interesting from a number of device applications perspectives, e.g. for demonstrating enhanced biochemical sensors in which the graphene acts as a high affinity surface functional layer for adsorbed species that may be probed by evanescent optical fields in the underlying silicon photonics device. The two-dimensional nature of graphene also leads to an optoelectronic band structure, the charge filling of which can be tuned by very low power electrostatic gating. In this case, the ‘Pauli blocking’ effect can alter the opacity of the material to incoming photons, providing the possibility of very fast (GHz) optical modulation or switching, which is likely to be of use in telecoms applications. Previous reports [17–20] of the in-plane linear absorption coefficient of graphene via integration with silicon photonics waveguide-based devices have yielded quite different results, suggesting that the specific transfer process and substrate interface quality in these studies may play some role in the variations observed. In this work, the spatial characterization of the Raman G and 2D peaks across a graphene-integrated silicon racetrack-type micro-ring resonator (MRR) is demonstrated using a mapping technique. Our approach is to investigate both the G and 2D peak frequencies, their relative integrated intensities and widths and correlate these with spatial position to elucidate the effect of the underlying silicon waveguide on graphene’s structural and optoelectronic properties at this interface.

## Methods/Experimental

The Si MRR devices in this study were fabricated in a commercial Si foundry (CEA-LETI, France) and comprise of strip-waveguides of 335-nm width, lithographically formed from commercial 220-nm silicon-on-insulator with a 2-μm-thick buried oxide layer. These waveguide dimensions, specifically, the relatively narrow waveguide width (compared with typical strip-waveguides), were selected to ensure good modal overlap with surface integrated graphene, post-transfer. Two ‘racetrack’-type MRR devices are studied, one in which the radial component is 10 μm and the other is 20 μm and both having identical 20-μm-long linear sections. Prior to graphene transfer, the devices were washed with acetone, isopropyl alcohol (IPA), de-ionized water and resist stripper (NMP: 1-methyl-2-pyrrolidone). This was followed by an oxygen plasma etch (for 40 s) immediately before transfer. The graphene was grown by chemical vapour deposition (CVD) on copper foils (Gratome-R-Cu, Bluestone Global Tech) and then transferred onto the pre-cleaned waveguides using a polymer-mediated wet transfer procedure [21]. The graphene was patterned, to ensure selective coverage of the MRR devices, using raster scan photolithography and oxygen plasma etching. In order to ensure as clean a sample as possible, a subsequent annealing treatment at 270 °C in a reducing atmosphere and acetone wash was applied leading to near complete removal of residual photoresist, as revealed by our optical images.

The Raman spectral mapping was performed at room temperature in back scattering configuration, using a Horiba LabRAM HR Evolution Spectrometer with a 600 g/mm grating. The scattering signal was collected confocally and detected with an integrated Peltier cooled charge coupled device (CCD) camera. The samples were excited by a 633-nm helium neon laser light, and mechanical movement of the sample during mapping was provided by a Marzhauser motorized microscope XYZ stage. The incident laser light was focused on the sample surface using a × 50 objective lens with numerical aperture of 0.75. In order to avoid laser heating, the laser power density on the sample was kept below 2 mW [22]. Raman maps were obtained for two different graphene-integrated silicon MRR devices, with radii of curvature *r* = 10 μm and 20 μm. The maps were obtained from a 120 × 120 point array with a step size between each point of 0.25 μm, and the precise frequency, intensity and width of the Raman G and 2D peaks were determined by fitting with Lorentzian line-shapes to the spectral peaks. From measurements of a single crystal silicon sample using the same instrument configuration (slit width, grating and excitation source), we estimate a spectral resolution from the bandwidth of the main Si scattering peak of 4.6 cm^−1^ or better.

## Results and Discussion

**Fig. 1:**
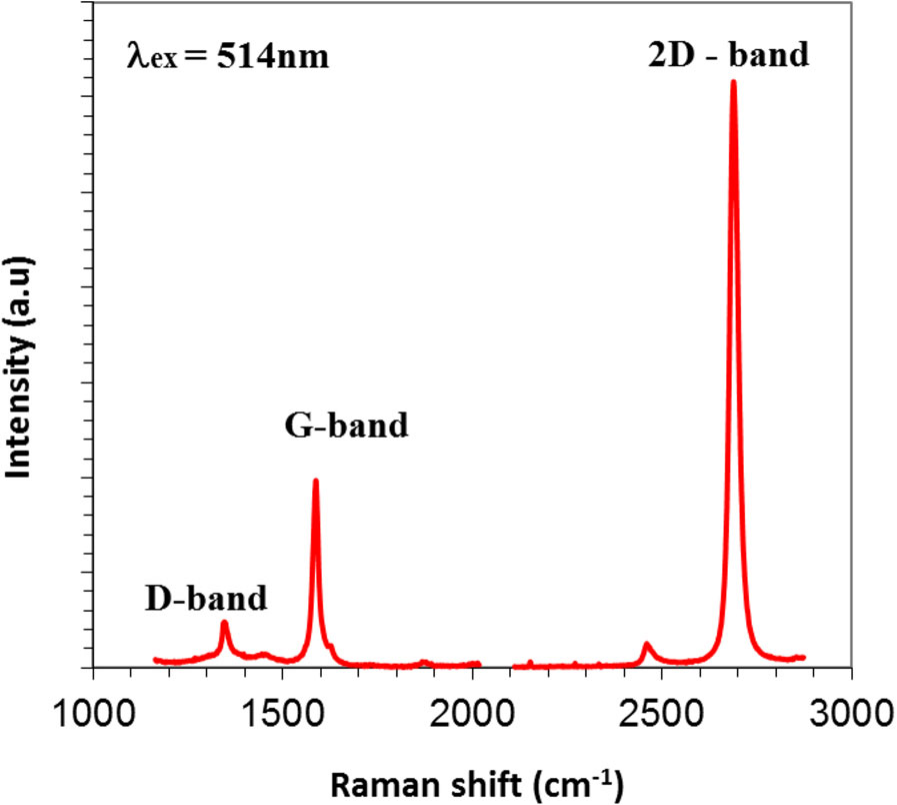
Single point Raman scattering spectrum (514-nm excitation) from which we infer the transfer of single layer graphene on the Si waveguide devices studied here as a result of the intense, symmetric 2D scattering mode and G peak frequency, ω_G_ ~ 1587 cm^−1^

**Fig. 2:**
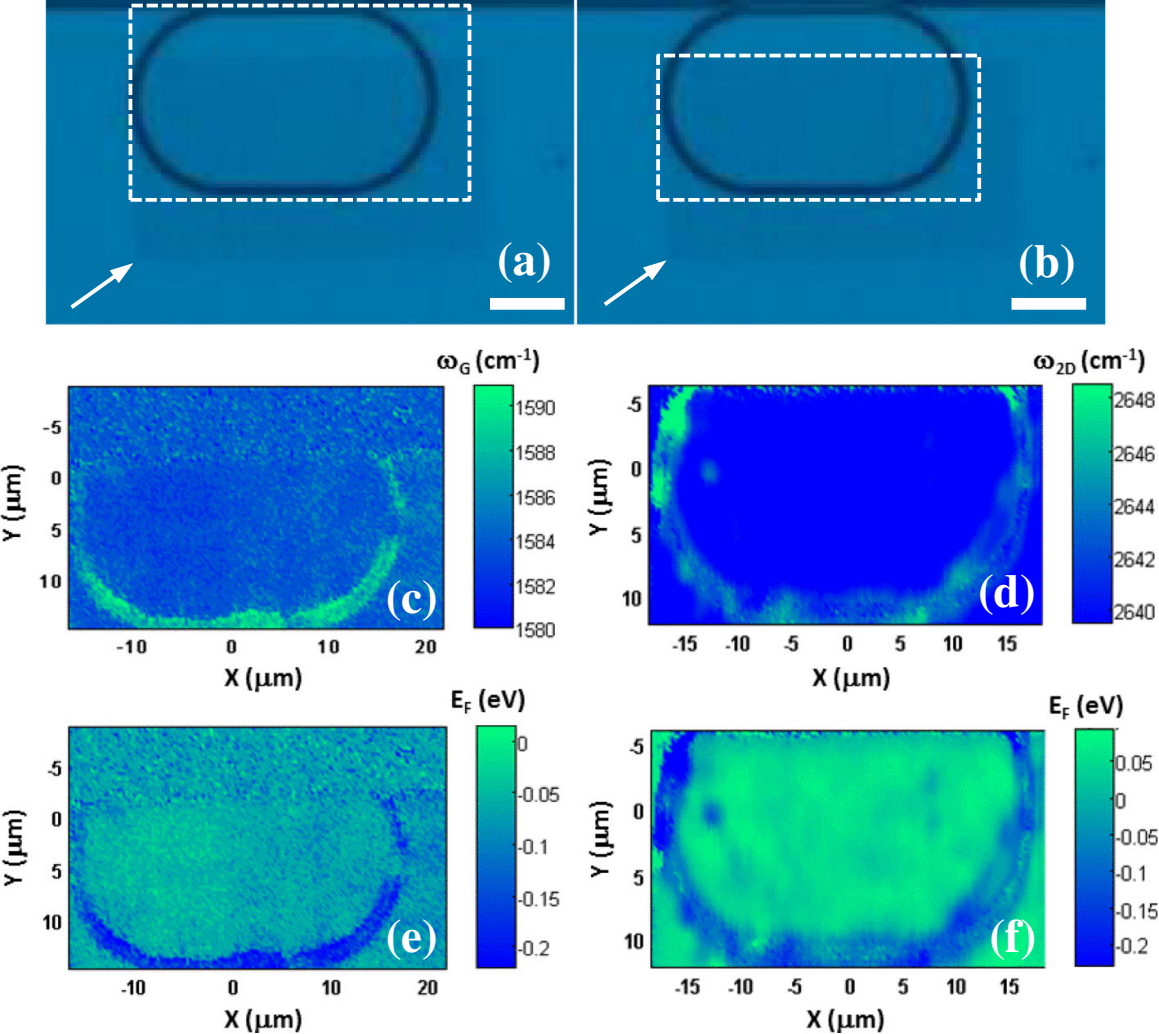
False colour optical image of the same graphene-coated Si MRR (*r* = 10-μm device) (scale bar = 10 μm) showing the different mapped regions (white dashed squares) for the **a** G and **b** 2D peaks, respectively. The graphene is revealed by the slightly darker contrast (with its bottom left hand corner indicated by the arrows). **c** and **d** show the corresponding peak positon and **e** and **f** the Fermi level maps, determined from Eqs. (1) and (2), respectively

In order to check if we had transferred the single layer graphene, prior to the Raman mapping study, we also measured the single point Raman scattering signal, Fig. 1, immediately after transfer (using a 514-nm Renishaw 1000 system). This spectrum reveals a weak Raman D peak indicating low structural disorder (reasonably high quality graphene); an intense (relative to the G peak), symmetric 2D scattering mode; and a G peak position of ~ 1587 cm^−1^. This combination of a relatively intense, symmetric 2D scattering peak and a G peak frequency close to the predicted value, ω_G_ (*n*) = 1581.6 + 11/(1 + *n* ^1.6^) where *n* is the layer number [23], confirms that the transferred graphene is indeed a single layer [24]. The optical image of the monolayer graphene-integrated MRR (*r* = 10 μm) is presented in Fig. 2a, b, and the mapped regions for the graphene G and 2D peaks are shown in Fig. 2a and Fig. 2b, respectively. Figure 2c, d are the resulting G and 2D peak positon maps, which reveal frequency up-shifts (of as much as ~ 11 and ~ 8 cm^−1^, respectively) where the graphene sits on top of the MRR waveguide structure relative to where it is suspended.

Shifted G and 2D Raman peaks can be associated with strain or doping or a combination of these in the graphene layer. However, in the low strain limit (where there is no splitting of the G peak), the strain-related shift of the 2D peak (∂*ω*_2*D*_/∂*ε*) is approximately six times that of the G peak (∂*ω*_*G*_/∂*ε*) [5]. That we observe broadly equivalent shifts of the G and 2D peaks where the graphene sits on the waveguide here indicates that the dominant cause of the shift is unlikely to be strain. On the other hand, the relative rate and direction of the G and 2D peak shifts with doping are highly specific to carrier type [25]. For both electron (*n*) and hole (*p*) doping, the frequency of the G peak always increases from the intrinsic value, meaning that a plot of G peak position with Fermi level is nearly symmetric about zero. However, for the 2D peak, whilst the frequency is up-shifted considerably for a moderate increase in *p*-doping level (~ 15 cm^−1^ for 3 × 10^13^ cm^−2^), it remains virtually unchanged from its intrinsic position up to an electron concentration of ~ 3 × 10^13^ cm^−2^, above which it down-shifts rapidly. This leads to a highly asymmetric curve for the 2D peak position with the Fermi level about zero. That we observe shifts that are both similar in magnitude and in the same direction for the G and 2D peaks strongly suggests that the graphene is moderately *p*-doped, where it sits on the waveguide, compared to where it is suspended. In order to quantify this effect, we used the following empirical relations (Eqs. (1) and (2)) to determine the approximate Fermi level shift from the Raman G and 2D peak shifts, after [25]:

1 $$ \left|{\mathit{\mathsf{E}}}_{\mathit{\mathsf{F}}}\right|\times \mathsf{41.5}=\Delta  {\omega}_{\mathit{\mathsf{G}}}=\omega \left(\mathit{\mathsf{G}}\right)-{\omega}_{\mathsf{0}}\left(\mathit{\mathsf{G}}\right) $$

2 $$ \left|{\mathit{\mathsf{E}}}_{\mathit{\mathsf{F}}}\right|\times \mathsf{31.5}=\Delta  {\omega}_{\mathsf{2}\mathit{\mathsf{D}}}=\omega \left(\mathsf{2}\mathit{\mathsf{D}}\right)-{\omega}_{\mathsf{0}}\left(\mathsf{2}\mathit{\mathsf{D}}\right) $$

where *ω*_0_(*G*) (=1580 cm^−1^ [26]) and *ω*_0_(2*D*) (=2640 cm^−1^ [9]) are the G and 2D peak positions, respectively, for unstrained, intrinsic graphene (for 633-nm excitation), *ω*(*G*) and *ω*(2*D*) are the G and 2D peak positions we have determined for each point in our maps and *E*_*F*_ is the Fermi level in units of eV. In Fig. 2e, f, we show the result of these calculations as Fermi level maps, derived from the data of Fig. 2c, d. These are broadly equivalent (as expected), indicating that the suspended graphene is intrinsic (*E*_*F*_ ~ 0) but that the hole concentration is increased (yielding a minimum value for *E*_*F*_ of approximately − 0.2 eV) where the graphene sits atop the waveguide structure. A similar analysis of an MRR with radius *r* = 20 μm (not shown here) gave a very similar result, indicating that the effect is not dependent on the waveguide geometry, rather that it is purely a material-dependent (substrate) doping effect. The source of this doping is almost certainly the result of locally trapped, static ad-charges at the interface between the silicon/SiO_2_ and graphene. The density of these ad-charges is known to be increased in samples which have received more aggressive cleaning treatments (such as the O_2_ plasma etch we have employed) [27]. Although this process provides a thoroughly clean interface (relatively free of contaminants), this damage can lead to oxygen-rich open-shell (dangling bond type) defects which are known to be effective charge carrier traps.

**Fig. 3:**
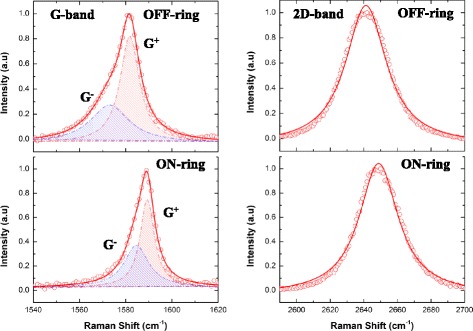
Representative graphene G (left) and 2D (right) averaged (*n* = 3) Raman scattering peaks (633-nm excitation) OFF (top) and ON (bottom) the underlying silicon MRR waveguide structure. Lines represent either double (G peak) or single (2D peak) Lorentzian fits to the data. The asymmetry in the G peak as a result of the lowering of the degeneracy of the in-plane *E*_2g_ optical phonon leads to distinct scattering modes, labelled G^+^ and G^−^ (in keeping with the convention used for carbon nanotubes)

Representative Raman scattering spectra (from the mapping) are shown in Fig. 3, revealing the up-shift in both the G and 2D peak frequency where the graphene sits on the underlying silicon MRR waveguide structure.

The 2D peak is well described (*R* ^2^ = 0.993) by a single, symmetric Lorentzian line-shape, a signature of single layer graphene [8]. We note that the fit to the 2D peak was only marginally improved using a Voigt function, which suggests only a small contribution to broadening from the instrument. No measurable change (beyond the standard error) was observed in the FWHM of the 2D scattering mode between ON- and OFF-ring data indicating an insensitivity of this to carrier concentration, consistent with previous observations [28].

The G peak, on the other hand, is rather asymmetric for both OFF- and ON-ring conditions and, as a result, is not well described by a single symmetric function. Rather, we found that it is best described (*R* ^2^ > 0.995) by a double Lorentzian line-shape, indicative of two distinct scattering processes. We note that the width of the main (G^+^) peak decreases by ~  25% (${\Gamma}_{\mathrm{OFF}}^{+}$ ~ 10 cm^−1^, ${\Gamma}_{\mathrm{ON}}^{+}$ ~ 7.5 cm^−1^) going from the suspended graphene to where it is supported by the MRR waveguide structure. This is consistent with current understanding and prior observations of the ‘stiffening’ of the graphene *E*_2g_ optical phonon, as a result of doping [8]. The second underlying scattering mode (G^−^), responsible for the asymmetry, also exhibits a significant decrease in width of ~ 35% (${\Gamma}_{\mathrm{OFF}}^{-}$ ~20 cm^−1^, ${\Gamma}_{\mathrm{ON}}^{-}$ ~ 13 cm^−1^) going from the suspended graphene to where it is supported by the MRR waveguide structure. Asymmetry in the graphene Raman G peak has previously been attributed to highly localized charge inhomogeneity within the laser probe area [28], i.e. on the sub-micron scale, and it has also already been observed when comparing Raman spectra of suspended graphene with that supported by a substrate [22]. Recent studies of graphene supported by nanostructured surfaces [29] have also revealed a multi-peak fine-structure in the G band, which was interpreted as being the result of extreme curvature or ‘wrinkling’, similar to what is observed in single-walled carbon nanotubes (SWCNTs). In this case, the doubly degenerate in-plane *E*_2g_ optical mode can be split between phonons along the nanotube axis, ${\omega}_G^{+}$, and those that are perpendicular to it, ${\omega}_G^{-}$, with the degree of splitting, $\Delta {\omega}_G={\omega}_G^{+}-{\omega}_G^{-}$, being a strong function of the nanotube size (i.e. degree of curvature), even in the absence of any externally applied strain [30]. G peak splitting has also been observed in graphene under uniaxial strain [5] and in isolated SWCNTs under hydrostatic pressure [31] where the curvature-sensitive lower frequency (G^−^) scattering mode itself can be broadened and even split when nanotubes buckle and collapse under high pressure loading. We note from fitting the graphene G band spectra here that both the frequency difference Δ*ω*_*G*_ and the line-width of the G^−^ mode (${\Gamma}_{\mathrm{OFF}}^{-}$) are greater for the suspended OFF-ring condition than for the ON-ring case. In the absence of any evidence (from the peak positions) for a global net strain, we speculate that this may be the result of a localized out-of-plane wrinkling in the suspended region, which is ‘smoothed’ out where the graphene is supported by the well-defined underlying sub-micron MRR waveguide structure, which would explain the smaller Δ*ω*_*G*_ and narrower G^−^ peaks we observe here.

We also examined the ratio of peak intensities, *I*_2D_/*I*_G_, which is known to be carrier concentration dependent, being maximum for the intrinsic case and decreasing continuously with increasing (both *n* and *p*) doping level, principally because of a quenching of the 2D mode with increasing carrier-phonon scattering [22, 32]. However, whilst we did observe a drop in *I*_2D_/*I*_G_, from ~ 3 where the graphene was suspended to ~ 2.5 on the waveguide structure, we note that this change is small relative to the degree of G peak shift we observe, when compared with other reports [28] for the same excitation laser wavelength (633 nm). It is worth pointing out though that in [28], there is a high degree of scatter in the data for *I*_2D_/*I*_G_ as a function G peak position, which appears to increase with excitation wavelength suggesting this alone may not be the most reliable indicator of absolute doping level, especially in the low doping limit.

**Fig. 4:**
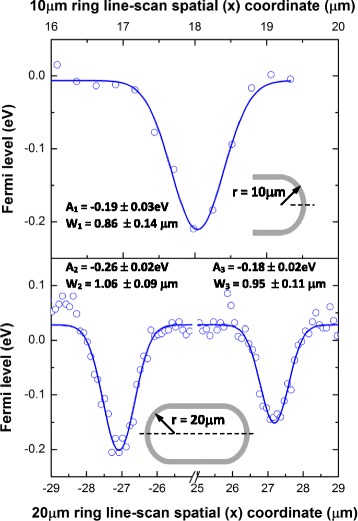
Graphene Fermi level determined (from $\surd \frac{A_G}{A_{2D}}$) as a function of spatial coordinate along line scans for (top) 10-μm- and (bottom) 20-μm-radius MRR devices (note the break in the bottom *x*-axis). Fitted (Gaussian) peak integrated areas and widths are shown for comparison along with where the line-scan data was taken on the devices

Analysis of the ratio of total integrated peak intensities, *A*_*G*_/*A*_2D_, which takes account of the peak widths as well as the variations in peak heights can be used to obtain the carrier concentration directly from Eq. (3) [22, 32]:

3 $$ \surd \frac{{\mathit{\mathsf{A}}}_{\mathit{\mathsf{G}}}}{{\mathit{\mathsf{A}}}_{\mathsf{2}\mathit{\mathsf{D}}}}=\mathit{\mathsf{C}}\left[{\gamma}_{\mathit{\mathsf{e}}-\mathit{\mathsf{ph}}}+\left|{\mathit{\mathsf{E}}}_{\mathit{\mathsf{F}}}\right|\mathit{\mathsf{f}}\left(\frac{{\mathit{\mathsf{e}}}^{\mathsf{2}}}{\varepsilon {\mathit{\mathsf{v}}}_{\mathit{\mathsf{f}}}}\right)\right] $$

where *C* is a constant; *e* is the electronic charge; γ_e-ph_ is the average electron-phonon scattering rate, previously determined in [32] to be ~ 33 meV; and ε (~ 3.9) is the dielectric constant of SiO_2_ [33], which is assumed to be present at the interface (as a native oxide layer) between the silicon and graphene. This yields *f*(e^2^/εν_*f*_) **~** 0.069 when ν_*f*_ is taken to be the electron velocity, 1.17 × 10^8^ cm/s. Our measurements indicate that $\surd \frac{A_G}{A_{2D}}$ is higher where the graphene sits atop the underlying silicon waveguide structure compared to the central suspended region, again supporting the hypothesis that the observed Raman spectral shifts are the result of a substrate doping effect. Figure 4 reveals the Fermi level we have determined from the ratio of integrated intensities of the graphene G and 2D modes and Eq. (3) as a function of position along spatial line scans made across the middle of the long section of the graphene-integrated MRR devices (for both 10- and 20-μm radii). The peak Fermi level shift coincides with where the graphene sits on the underlying silicon waveguide structure and is ~ 0.2 eV, in agreement with what we have determined from the peak shifts and that previously determined for a back-gated graphene field effect transistor [17]. It is worth pointing out that, despite the different device geometries we have studied, which leads to a larger region of suspended graphene over the 20-μm radius MRR structure compared with the 10-μm radius structure (~ 54-μm suspended graphene compared with ~ 36 μm, respectively), the local spatial doping pattern is virtually identical, as revealed by the Gaussian fits in Fig. 4.

Converting the Fermi level we have determined to a carrier concentration, *n* through Eq. (4) [33] yields a peak value for *n* ~ 3 × 10^12^ cm^−2^ on the MRR structure, which is in generally good agreement with previous reports [26]:

4 $$ \mathit{\mathsf{n}}={\left(\frac{{\mathit{\mathsf{E}}}_{\mathit{\mathsf{F}}}}{\hslash {\nu}_{\mathit{\mathsf{F}}}}\right)}^{\mathsf{2}}/\pi $$

**Fig. 5:**
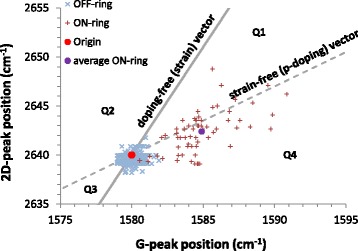
G-2D correlation plot showing data for three line-scan measurements across the graphene-integrated MRR. The red crosses are points taken where the graphene sits ON the MRR structure with the purple dot representing the average of these coordinate values and the blue crosses where the graphene is suspended across the MRR (OFF the underlying structure). The red dot is the unstrained, intrinsic coordinate value for graphene with 633-nm laser excitation, which defines the origin. The dashed line denotes the strain-free (*p*-doping) vector with ∆ω_2D_/∆ω_G_ ~ 0.7, and the solid line denotes the doping-free (strain) vector with ∆ω_2D_/∆ω_G_ ~ 2.2, after [34]

Finally, we examined the correlation between the G and 2D peak positions from our measured data (from three line-scans) in a so-called vector decomposition plot, introduced by Lee et al. [34], Fig. 5.

Representing the data in this type of plot enables us to determine to what degree the peak shifts may be influenced by strain. This is based on the fact that the rates of variation in peak position ratios for strain (∆*ω*_2*D*_/∆*ω*_*G*_ ~ 2.2) are very different for those associated with doping (∆*ω*_2*D*_/∆*ω*_*G*_ ~ 0.7) [34]. Any coordinate point in the G-2D space can therefore be decomposed into strain and, specifically, *p*-type doping vectors. With increasing tensile strain *or p*-doping, the *ω*_*G*_, *ω*_2*D*_ coordinate values will move from the origin (intrinsic, unstrained position), either along the doping-free (strain) or strain-free (*p*-doping) lines, respectively. The G-2D coordinate space is divided into four quadrants, Q1–Q4 by these strain and doping vectors, and so any significant deviation of the coordinate data from these lines, say into region Q1 (Q4), would indicate that the peak shifts are the result of a combination of compressive (tensile) strain *and p*-doping. Scattering of data within Q2 and Q3 is forbidden because both *n-* and *p*-doping manifests only in increases in the G peak position.

We define the intrinsic, unstrained graphene peak frequency coordinate as the origin (red dot) [9, 26] and indicate the strain-free (*p*-doping) vector (dashed line) and doping-free (strain) vector (solid line), after [31]. Data for three different line-scans is scattered around the origin for the OFF-ring and along the strain-free (*p*-doping) vector for the ON-ring with the average ON-ring coordinate value (purple dot) being (1584.9, 2642.4). The increased scatter for the ON-ring data along the strain-free line indicates a greater range of doping levels detected from the relative peak shifts, likely because of the uncertainty in probing a highly localized substrate doping effect produced by the underlying, sub-micron waveguide width, compared with the probe laser spot size (> 1 μm). Despite the apparent scatter in the data, into both Q4 and Q1, we discount any significant global strain effects because the average ON-ring coordinate lies so close to the strain-free line. We suggest that the peak shifts we observe are only due to silicon substrate induced hole-doping and the average ON-ring G-2D coordinate confirms this to be in the range of (2 to 3) × 10^12^ cm^−2^.

## Conclusions

In summary, monolayer CVD graphene was integrated with silicon waveguide-based MRR photonic devices. Frequency shifts and integrated intensities of the characteristic graphene Raman G and 2D peaks were determined for mapped regions, and these indicate a Fermi level ‘pinning’ where the graphene sits on the Si MRR structure as a result of unintentional hole-doping from the underlying silicon/SiO_2_ waveguide (substrate doping effect). The data for the suspended region reveals no measurable distinction from intrinsic graphene, but for the supported region, a maximum down-shift of the Fermi level of ~ 0.2 eV is determined, which corresponds to a peak hole concentration of ~ 3×10^12^ cm^−2^. An asymmetry in the Raman G peak, which varies according to whether the graphene is suspended or supported, indicates a combination of doping-induced ‘stiffening’ and lifting of the degeneracy of the *E*_2g_ optical mode. These effects should be taken into account when graphene is combined with silicon photonics platforms, certainly when attempting to use such platforms to determine graphene’s characteristic properties and for optimization of future graphene-integrated silicon photonics devices, such as optical modulators and sensors.

## Abbreviations

CCD: Charge coupled device
CEA-LETI: Commissariat à l’energie et aux energies alternatives–laboratoire d’électronique des technologies de l’information
CMOS: Complementary metal oxide semiconductor
CVD: Chemical vapour deposition
DR: Doubly resonant
FWHM: Full width at half maximum
MRR: Micro-ring resonator
NMP: N-Methyl-2-pyrrolidone
Si: Silicon
SiO_2_: Silicon dioxide
SWCNT: Single-walled carbon nanotube
